# Supplementary material for: Revealing principles of autonomous thermal soaring in windy conditions using vulture-inspired deep reinforcement-learning
Source: Nat Commun. 2024 Jun 10;15:4942. doi: 10.1038/s41467-024-48670-x (PMC11164704; doi:10.1038/s41467-024-48670-x)
Supplement: Supplementary file 1 — Supplementary Information [file 41467_2024_48670_MOESM1_ESM.pdf]

Supplementary Information

Revealing principles of autonomous thermal soaring in windy conditions using vulture-inspired deep reinforcement-learning

Yoav Flato, Roi Harel, Aviv Tamar, Ran Nathan, and Tsevi Beatus
Nature Communications https://doi.org/10.1038/s41467-024-48670-x (2024)

Supplementary Note 1: Aerodynamic force model and glider parameters

Following Beeler *et al.*[1], the environment is based on a 3 degrees-of-freedom (DOF) simulation of a point-mass glider governed by the equations of motion (Eq. 2, main text). The aerodynamic forces: lift L, drag D and side force C, are:

$$L(V,\alpha) = \frac{1}{2}\rho S C_L(\alpha)V^2 \tag{S.1}$$

$$D(V,\alpha,\beta) = \frac{1}{2}\rho S C_D(\alpha,\beta)V^2 \tag{S.2}$$

$$C(V,\beta) = \frac{1}{2}\rho S C_C(\beta)V^2, \tag{S.3}$$

where the forces are functions of the velocity V, the aerodynamic coefficients $C_L(\alpha)$,$C_D(\alpha,\beta)$,$C_C(\beta)$, the wing surface area S, the air density ρ. The force coefficients are functions of the angle-of-attack α and the sideslip angle β, and depend also on the glider's mass, wingspan and wing aspect ratio (Supplementary Table 1.1). Following Beeler *et al.*[1], To account for the effects of wind on the glider, the full aerodynamic model calculates the forces in the wind frame-of-reference and then transforms these forces to the world frame-of-reference for solving the equations of motion.

Symbol	Name	Value	Units
AR	aspect ratio	16	–
l	wingspan	2.5	m
S	wing area	0.39	m^2
m	mass	5	kg

TABLE 1.1. **Glider parameters.** The glider parameters used in the simulation are based on Beeler *et al.*[1].

Supplementary Note 2: An upper bound for the thermal climb rate

One way to evaluate a policy of an agent in our system, would be to compare the policy climb rate performance (mean v_z) in a given environment to the maximum climb rate possible under the same conditions. Hence, we estimated an upper bound for the climb rate, as described below.

We assume that an optimal trajectory has constant rotation radius in the wind frame of reference and, therefore, constant control parameters (σ,α,β) in that frame. Note that to keep these control parameters constant in the wind frame of reference (under any wind, horizontal or vertical), an agent would need to modulate them in the body frame of reference. Further, we assume β=0 as used in our nominal agent.

First, for each pair of (σ,α) we analytically solve the equations of motion (Section IV B, main text) in steady state and find the values of V_{ss}, γ_{ss} and R_{ss}, where the latter is the trajectory radius. Second, we define, d, the distance between the center of the trajectory and the thermal center, and use numerical optimization to find (σ,α,d) that result in maximum climb rate v_z in the world frame of reference. These two stages are detailed below.

In steady state of Eq. 2 (main text) the velocity and glide angle are constant:

$$\dot{V} = 0 = -\frac{1}{m}D(V_{ss},\alpha,\beta) - g\sin\gamma_{ss}$$
$$\dot{\gamma} = 0 = \frac{1}{mV_{ss}}\Big(L(V_{ss},\alpha)\cos\sigma + C(V_{ss},\beta)\sin\sigma\Big) - \frac{g}{V_{ss}}\cos\gamma_{ss} \tag{S.4}$$

Assuming β=0, we get:

$$D(V_{ss},\alpha,0) = -mg\sin\gamma_{ss}$$
$$L(V_{ss},\alpha)\cos\sigma = mg\cos\gamma_{ss} \tag{S.5}$$

By dividing these two equations and rearranging, we obtain an expression for the steady state glide angle:

$$\gamma_{ss}(\sigma,\alpha) = \arctan\left(-\frac{C_D(\alpha,0)}{C_L(\alpha)cos(\sigma)}\right). \tag{S.6}$$

We then substitute the definition of $D(V,\alpha,\beta)$ from eq. (S.3) into eq. (S.5) to get V_{ss}:

$$V_{ss}(\sigma,\alpha) = \sqrt{-\frac{2mg\sin(\gamma_{ss}(\alpha,\sigma))}{C_D(\alpha,0)S\rho}}. \tag{S.7}$$

In this steady state of circular motion in the wind frame of reference, the radial force due to the tilted lift vector is equal to the radial acceleration: $L(V_{ss},\alpha)\sin(\sigma) = mV_{ss}^2/R_{ss}$. To find R_{ss} from this equation, we substitute $L(V_{ss},\alpha)$ from eq. (S.3):

$$R_{ss}(\sigma,\alpha) = \frac{2m}{C_L(\alpha)S\rho\sin(\sigma)}. \tag{S.8}$$

Note that because $L{\sim}V^2$, then R_{ss} is independent of V_{ss}. The steady state climb rate in the wind frame is:

$$v_z^{wind}(\sigma,\alpha) = V_{ss}(\sigma,\alpha)\sin(\gamma_{ss}(\sigma,\alpha)) \tag{S.9}$$

Next, for each combination of (σ, α, d), we calculate the average climb rate v_z in the world frame of reference. This climb rate consists of two terms:

$$v_z(\sigma,\alpha,d) = v_z^{\mathrm{wind}}(\sigma,\alpha) + v_z^{\mathrm{thermal}}(\sigma,\alpha,d). \qquad \text{(S.10)}$$

The first term is given in eq. (S.9) at steady state, and the second term is the vertical velocity of the wind frame itself due to the thermal updraft averaged across the agent's trajectory. Each pair of control parameters (σ, α) defines a value of R_{ss} (eq. (S.8)). Together with d, and relying on the radial symmetry of the thermal, we numerically calculate the mean thermal updraft along one rotation period $v_z^{\mathrm{thermal}}(\sigma,\alpha,d)$:

$$v_z^{\mathrm{thermal}}(\sigma,\alpha,d) = \frac{1}{T}\int_0^T w(r,z)dt, \qquad \text{(S.11)}$$

where T is the rotation period, $w(r,z)$ is the thermal updraft velocity (Eq. 3, main text), $r = r(d, R_{\mathrm{ss}}, t)$ is the glider distance from the thermal center, and z is the glider altitude. We assume constant altitude of 500m because we characterize the optimal updraft at this altitude and because the thermal updraft profile does not change much with altitude (main text Figures 1c,d).

Finally, using numerical optimization on (σ, α, d), we find that under our simplifying assumptions the upper bound the the climb rate in the world frame of reference is obtained for $\alpha \approx 13.3°$, $\sigma \approx 31.5°$, $d = 0$ (*i.e.*, flying directly around the thermal center) and $R_{\mathrm{ss}} = 28.2\,\mathrm{m}$. The upper bound is:

$$v_z^{\mathrm{optimal}} = 0.72\,\mathrm{m/sec} \qquad \text{(S.12)}$$

Supplementary Note 3: Hyper-parameters search

We used a Bayesian method for hyper-parameter search[2] to optimize the following hyper-parameters of our RL system: the learning algorithm (DDPG[3] or PPO[4]), learning rate, and the NN architecture (hidden layer size and number of hidden layers) for both actor and critic. Each combination of hyper-parameters, was tested on a model problem of thermal soaring under u=1 m/sec given 10^6 time steps (~5,000 thermals) to converge.

The optimization results are summarized in Supplementary Figure 3.1. The chosen algorithm was DDPG with learning rate of 10^{-3}. The actor NN architecture was: 2 hidden layers of 200 neurons each and ReLU activation, and tanh activation for the output layer. The critic NN architecture was the same, without an activation function on its (scalar) output. In this parameter scan, the PPO algorithm achieved similar results but with larger networks of 500 neurons in 2 hidden layers.

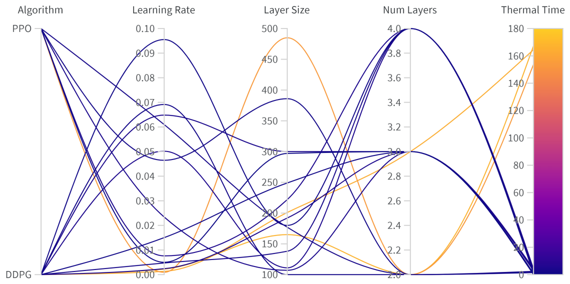

FIG. 3.1. **Bayesian Hyper-Parameter Search Results.** This scheme includes of five columns: four for each hyperparameter and the rightmost one for the agent's performance in terms of its average thermal time. Each continuous line represents a single run of 10^6 timesteps, indicating the values of the four hyperparameters and gliding performance. The lines are color-coded by gliding performance.

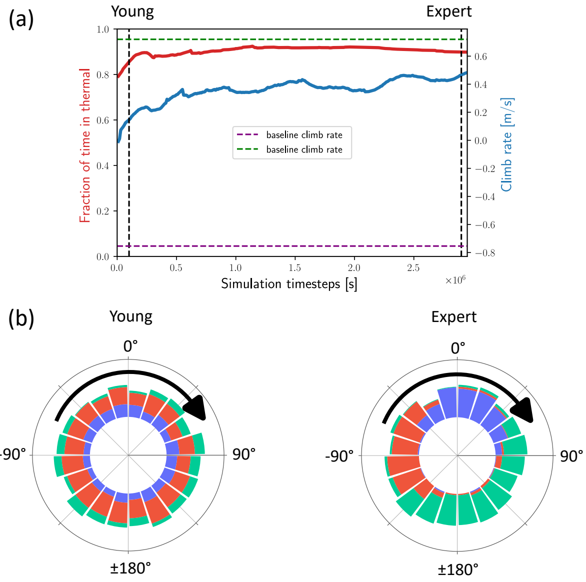

FIG. 4.1. **NN neural clusters during the learning process of reduced-state agent.** **(a)** Performances values during the learning process. The high performance values observed in the beginning of the run are attributed to the fact that this learning process started from pre-trained agents. **(b)** Distribution of the clusters of each agent as a function of θ.

Supplementary Note 4: Clustering analysis of agents with a reduced state representation

To verify that the activity distribution of the NN clusters in θ was not a trivial outcome of having θ as an implicit state variable, we trained an agent whose state did not include θ. Instead, the state included $\{V, v_z, \sigma\}$ and the agent was trained on one value of u=1 m/sec. The agent with this reduced state-representation achieved stable soaring and exhibited similar clustering characteristics that evolved to be θ-dependent with the agent's 'age' (Supplementary Figure 4.1).

SUPPLEMENTARY REFERENCES

[1]S. C. Beeler, D. D. Moerder, and D. E. Cox, "A flight dynamics model for a small glider in ambient winds," Tech. Rep. (2003).

[2]S. Falkner, A. Klein, and F. Hutter, "BOHB: Robust and efficient hyperparameter optimization at scale," in *International Conference on Machine Learning* (PMLR, 2018) pp. 1437–1446.

[3]T. P. Lillicrap, J. J. Hunt, A. Pritzel, N. Heess, T. Erez, Y. Tassa, D. Silver, and D. Wierstra, "Continuous control with deep reinforcement learning," arXiv preprint arXiv:1509.02971 (2015).

[4]J. Schulman, F. Wolski, P. Dhariwal, A. Radford, and O. Klimov, "Proximal policy optimization algorithms," arXiv preprint arXiv:1707.06347 (2017).
