# Supplementary material for: Revealing principles of autonomous thermal soaring in windy conditions using vulture-inspired deep reinforcement-learning
Source: Nat Commun. 2024 Jun 10;15:4942. doi: 10.1038/s41467-024-48670-x (PMC11164704; doi:10.1038/s41467-024-48670-x)
Supplement: Supplementary file 3 — Description of additional supplementary files [file 41467_2024_48670_MOESM3_ESM.pdf]

<u>Description of Additional Supplementary Files</u>

Supplementary Movie 1 : A representative trajectory of the nominal agent soaring in a thermal under horizontal wind of 2.4 m/s.

Supplementary Movie 2 : Similarly, a trajectory of the same agent soaring under horizontal wind of 3 m/s.
